# Hearing loss and psychosocial outcomes: Influences of social emotional aspects and personality

**DOI:** 10.1371/journal.pone.0304428

**Published:** 2024-06-12

**Authors:** Kayla Cormier, Christine Brennan, Anu Sharma

**Affiliations:** Department of Speech Language and Hearing Sciences, University of Colorado Boulder, Boulder, CO, United States of America; Universiti Malaya Fakulti Perubatan: University of Malaya Faculty of Medicine, MALAYSIA

## Abstract

The aim of this study was to examine the effects of social isolation, loneliness, anxiety, depression, higher stress, and memory complaints interacting with personality traits as a function of hearing loss. Personality traits have previously been shown to correlate with anxiety and depression, as well as hearing loss, suggesting an effect of personality on the relationship between social emotional outcomes and hearing loss. A cross-sectional survey including validated screening measures was anonymously administered. Measures included personality (Big Five Index-2 Extra-Short Form), stress (Perceived Stress Scale), anxiety and depression (Patient Health Questionnaire for Depression and Anxiety), loneliness (Three-Item Loneliness Scale), social engagement (Lubben Social Network Scale-6), and self-perceived memory complaints (Subjective Cognitive Function). Eight hundred and ninety-one responses were obtained from adults between the ages of 18 and 90 years old (M = 50 years old). Hearing loss was positively correlated with subjective memory complaints only when not accounting for demographic variables, personality traits, psychosocial outcomes, and social emotional outcomes. There were additive effects of hearing loss and negative emotionality on increases in memory complaints in those who self-identified as maybe having hearing loss. Higher degrees of hearing loss also increased loneliness, with greater hearing loss negating the positive correlation between extraversion and social engagement. Overall, our results suggest that hearing loss significantly interacts with personality traits and other social emotional measures. Our results suggest that the impact of hearing loss on memory complaints, social isolation, and loneliness may differ across patients with hearing loss in comparison with those who think they maybe have hearing loss. Information from this study may provide insights for hearing healthcare and mental healthcare professionals to better serve persons with hearing loss who may require additional support or interventions.

## Introduction

Age related hearing loss affects an estimated 1.5 billion persons globally, making it one of the most common chronic health conditions in adults [1]. Untreated hearing loss is associated with cognitive decline and has recently been identified as a major independent and possibly modifiable risk factor for dementia [2, 3]. Although the relationship between hearing loss and cognitive decline has been demonstrated, the mechanism behind this relationship is still in question. Three of the theories that have been proposed have potentially differing implications for the expected outcomes of hearing treatment. A current theory suggests that listening effort due to degraded speech input from hearing loss increases a person’s cognitive load, as the individual is constantly utilizing top-down cognitive processing to make up for the poor sensory input. As a result, the cognitive load theory (see also the information degradation theory or Framework for Understanding Effortful Listening) suggests listening effort secondary to hearing loss results in a chronic taxing of cognition, specifically attention, memory, and executive functioning, resulting in lower global cognitive abilities in people with hearing loss compared to age matched peers [4–8]. This theory posits that the effects of hearing loss on cognition are temporary and may resolve with hearing treatments [7, 8]. The common cause theory suggests that the association between hearing loss and cognition is the result of another factor that causes both decreases in hearing and decreases in cognition simultaneously [7–9]. For example, cerebrovascular disease could result in changes in both cognition and hearing abilities [2]. This theory would not predict that hearing treatments would influence cognition [7, 8]. Finally, the cascade hypothesis suggests that auditory deprivation occurring not only due to the hearing loss, but also other hearing loss consequences such as social isolation, results in changes in the brain such as cortical re-allocation, deafferentation, or atrophy [7–10]. These cortical changes in turn can have a negative impact on cognition. However, reversal of these cortical changes by hearing treatments should predict increases in cognition with hearing treatments [8].

Hearing loss has not only cognitive consequences but may result in various physical and social emotional changes [11–14]. Therefore the relationship between hearing loss and cognitive decline may be mediated by a decline in social emotional wellbeing [9]. Research has shown that hearing loss is correlated with a higher risk of both social isolation and loneliness [15, 16]. Recent meta-analyses have concluded that hearing loss is associated with depression [17, 18]. Hearing loss has also been found to be correlated with increases in stress and anxiety in addition to depression [12, 14, 19, 20]. Jayakody et al. [12] found that while hearing loss severity was associated with increases in emotional loneliness, depression and stress were associated with increases in social loneliness while hearing loss severity was positively associated with depression and stress. Therefore, a complex relationship may be present between the effects of hearing loss on psychosocial outcomes and the down-stream effects of those psychosocial outcomes on other mental health outcomes. Furthermore, Kiely et al. [21] demonstrated that while hearing loss severity was associated with increases in depression, accounting for activity engagement fully explained the relationship between hearing loss and depression. These studies suggest that the presence of hearing loss with changes in psychosocial well-being could interact in an intricate manner. Additionally, the COVID-19 pandemic highlighted the association between hearing loss and social emotional well-being. A recent survey during the COVID-19 pandemic found that patients with hearing loss fit with cochlear implants (CI) reported less diversity in listening environments, less interaction with people, and more loneliness than before the pandemic [22]. Considering these findings, Timmer et al. [23] has called for hearing healthcare providers to adopt individualized treatment plans for hearing loss patients that address social-emotional well-being. Specifically, these authors suggest the use of anxiety, depression, and social isolation questionnaires to tailor hearing loss interventions.

In fact, treatments for hearing loss could positively impact social emotional wellbeing. The use of hearing aids is reported to improve social support, decrease the odds of reporting psychological distress, and decrease odds of reporting depression in comparison to those with untreated hearing loss [24–26]. Cochlear implantation also results in decreases in depression [26]. However, not all studies have demonstrated improvements in all areas of wellbeing with hearing aid use and researchers have suggested that these inconsistent findings confirm a need for more research into the associations of hearing treatments on social emotional wellbeing [9, 25]. Additionally, treatment with hearing aids and CI may vary in impact. In studies that have compared CI users and hearing aid users, CI users demonstrated significantly higher depression and loneliness scores pre-treatment than hearing aid users [26, 27]. After treatment, depression scores remained significantly lower for CI users throughout the first year of CI use but hearing aid users only showed significantly lower depression scores over the first 6 months of hearing aid usage [26]. Therefore, measures of social-emotional well-being (anxiety, depression, and social isolation) could be helpful for clinicians if utilized pre-and post-hearing loss treatment to identify the need for new interventions and/or goals [23]. In terms of cognition, both hearing aid and CI use have been associated with improvements in multiple aspects of cognition, including executive functioning, working and spatial memory, processing speed, visual learning, and visual attention in individuals with hearing loss [28–32]. Yet, other research findings suggest no significant improvements in cognition with hearing treatments [33–35]. Overall, the impacts of hearing treatments on psychosocial and social-emotional outcomes are currently mixed and warrant further investigation.

Personality traits can also affect both social emotional outcomes and cognition. Nikčević et al. [36] found that higher levels of extraversion, agreeableness, conscientiousness, and openness as measured by the Big Five Inventory-10 [37], negatively correlated with general anxiety and depression scores. The Big Five Inventory-10 is an established tool to examine personality traits focused on five factors including neuroticism, extraversion, openness to experience, agreeableness, and conscientiousness. This inventory was created by abbreviating a previous personality inventory known as the standard BFI (Big Five inventory) which had 44 items [38]. In general, studies suggest higher scores in neuroticism have been associated with worse cognitive performance, while higher scores on openness and conscientiousness are related to better cognitive performance [39, 40]. Sutin et al. [39] found that greater agreeableness was also related to better cognitive performance, while extraversion did not seem to have much of an impact on cognition. Kendler and Myers [41] established a negative correlation between conscientiousness and depression, as well as a positive correlation between neuroticism and depression.

Very few studies have examined the effect of personality traits in hearing loss patients. In terms of self-rated hearing loss, the rate of decline in extraversion, openness, agreeableness, and conscientiousness is faster in those reporting worse hearing abilities measured across four years [42]. In fact, only self-reported hearing loss was found to be associated with increased rates of decline in extraversion in a study of older adults over six years even when including self-reported general health or number of diagnoses in the analysis of this change [43]. The rate of increases in neuroticism were also faster in those noting worse hearing [42]. However, among hearing aid users, greater degrees of hearing loss were not related to differences in personality traits [44]. Lower scores in extraversion, openness, agreeableness, and conscientiousness, with higher scores in neuroticism, predicted worsening hearing four years later, as well [42]. On the other hand, higher scores on extraversion, openness, agreeableness, and conscientiousness were associated with lower risk of hearing loss progression [45]. In terms of hearing aid treatment, individuals with lower scores in neuroticism and openness were more likely to pursue hearing aids [44]. Additionally, higher scores in neuroticism were found to be related to poorer hearing aid outcomes including reporting residual difficulties with hearing aid use [46, 47]. Cox et al. [47] found personality affects self-reported outcomes on commonly used hearing aid questionnaires; however, following the creation of three factors from an exploratory factor analysis of these hearing aid questionnaires and inclusion of pre-fitting reports about hearing problems, expectations for hearing aids, and sound aversion, personality was no longer significantly or independently related to these measures. This underscores the importance of controlling variables when examining hearing loss, hearing treatments, and personality.

Largely, there is very little information regarding the relationship between personality, social wellbeing, and cognition in hearing loss patients. Such information may be valuable in allowing us to better understand the association between hearing loss and cognition, such as memory complaints, as well as better inform practices of both mental health professionals and hearing healthcare professionals to possibly deliver more customizable experiences to patients with hearing loss. To this end, our exploratory study aimed to examine the relationships between hearing loss, personality, and wellbeing. Given that clinical audiology patients present with limited social engagement, loneliness, anxiety, depression, stress, and memory concerns, we expected these variables to be related to hearing loss in a complex manner. Furthermore, previous studies have demonstrated differences in personality traits of those who obtain and benefit from hearing loss treatments [44, 47]. Yet, few studies have explored how all these factors interact in individuals with hearing loss. Therefore, this study aimed as a first step to identify and better understand the relationships between hearing loss, loneliness, social isolation, stress, anxiety, depression, and memory complaints.

## Materials and methods

### Study design

A cross-sectional, anonymous survey design was utilized for this study allowing for an investigation of a wide array of individuals. Inclusion criteria included residence within the United States and being 18 years of age or older. The survey was administered online via Qualtrics from February 8, 2021, to June 12, 2021. This data was collected during the COVID-19 pandemic, as such a question regarding COVID-19 vaccination status was added on March 22, 2021, with the majority (61%) of the survey respondents answering this question. The survey was distributed through Research Match and via social media, which included both group sites (hearing aid tracker, agency on aging, local city/town pages, etc.) and personal social media pages. No personally identifiable information was collected, questions within the survey were not randomized, and participants could only submit one response. Two validation questions were utilized in which the participant was instructed to select a particular response. Participants who did not select the indicated response for these questions were excluded from analysis. This survey was approved by the University of Colorado Boulder Institutional Review Board (IRB). All participants included in this study were first presented with an explanation of the study, after which they were asked to indicate if they consented to continue the study. Those who selected not to consent to the study terminated the questionnaire at that time and were thanked for their participation.

Closed ended questions were utilized for a demographics section including age, gender, employment status, education, marital status, number of individuals in the household, pets in the household, COVID-19 vaccination status, frequency of social media use, news consumption, the presence of tinnitus, self-reported hearing loss, use of amplification, and severity of hearing loss. Questions relating to COVID-19 vaccination status and pets in the household were included in this study due to the potential impact these factors could have on psychosocial and social emotional measures during the pandemic. Therefore, both of these measures were included in this analysis. For self-reported hearing loss, respondents could select that they did not have hearing loss, maybe had hearing loss, or had hearing loss. Previous research has shown self-reported hearing loss to be sensitive to objective measures of hearing thresholds [48–50]. For those who self-reported having a hearing loss they answered more detailed questions about the hearing loss such as which ear and/or ears had hearing loss, the use of hearing loss treatment devices, duration of device use, and severity of hearing loss. Validated questionnaires were employed in this study to cover the domains of psychosocial measures (stress, loneliness, memory complaints), social emotional measures (anxiety, depression, and social isolation), and personality. The questions used in this survey can be viewed in S1 Appendix.

### Psychosocial measures

The Three-Item Loneliness Scale was utilized to capture loneliness in this study. The Three-Item Loneliness scale is measured on a Likert scale from 1 (hardly ever) to 3 (often) indicating greater loneliness for higher scores [51]. This questionnaire was adapted from the longer Revised UCLA Loneliness Scale by selecting the three questions from Revised UCLA Loneliness scale that loaded onto the first factor of both an exploratory and confirmatory factor analyses. The Three-Item Loneliness scale is strongly, positively correlated with the Revised UCLA Loneliness Scale and the internal reliability, as measured with Cronbach’s Alpha, of this scale has been noted to be 0.72. Thus, the Three-Item Loneliness Scale has demonstrated good internal consistency, as well as good discriminant and convergent validity as a measure of loneliness [51].

Stress was measured using the short four item Perceived Stress Scale (PSS-4) using a Likert scale from 0 (never) to 4 (very often) [52]. The PSS-4 was adapted from a longer version with 14 questions. The four questions selected for the PSS-4 have been shown to load onto one factor and demonstrate an internal reliability alpha coefficient of 0.72 [52].

To measure memory complaints, we employed the Subjective Cognitive Function (SCF) questionnaire using six yes/no questions with 1-point given for each yes response to the six included memory concerns. These questions are the same as those use by previous investigations of cognitive function and decline [53]. A study that examined whether physical activity was associated with late-life cognitive function measured subjective cognitive function using six yes/no questions about recent changes in the ability to remember recent events, remember a short list, remember things from one second to the next, understand or follow spoken instructions, follow a group conversation or plot of a show, and find one’s way on familiar streets. Two other studies [54, 55] examined the relationship between cognitive function and hearing also utilizing the same SCF questions as Fondell et al. [53]. These previous studies demonstrated a relationship between severity of hearing loss and SCF in both men and women [54, 55].

### Social emotional measures

A 6-item Lubben Social Network Scale (LSNS-6) was administered with a Likert scale from 0 (none) to 5 (nine or more). Lower LSNS-6 scores indicate more social isolation, with the highest possible score being 30. The LSNS-6 includes three social engagement questions related to family and three questions related to friends [56]. Factor analysis of the LSNS-6 has demonstrated that each of these three items load heavily on to their respective subscales. The LSNS-6 also has been shown to have good discriminate validity and internal reliability with Cronbach’s Alpha coefficients of 0.83 having been reported for this scale indicating satisfactory reliability for this outcome measure [57]. In this study the subscales were not analyzed separately with only the total score being employed as both an independent and dependent variable.

The Patient Health Questionnaire-4 (PHQ-4), which is also a 4-item form, was used to examine anxiety (first 2 questions) and depression (last 2 questions). The PHQ-4 utilizes a Likert scale from 0 (never) to 3 (nearly every day) with a score greater than or equal to three on the two questions in each domain suggestive of anxiety and/or depression [58]. Factor analysis has confirmed that the respective items load on the two subscales of anxiety and depression. Additionally, a good internal reliability with a Cronbach’s Alpha of 0.78 has been noted for the PHQ-4, confirming the PHQ-4’s validity and reliability as a measure [59].

### Personality

The Big Five Inventory–2 Extra-Short Form (BFI-2-XS) was used to examine personality across the categories of extraversion, agreeableness, conscientiousness, negative emotionality, and open-mindedness. The BFI-2-XS is a 15-question measure that is scored using a Likert scale from 1 (disagree strongly) to 5 (agree strongly). Personality traits are measured separately and the separate totals from each personality trait were used in all analyses in this study [60]. The Big Five personality traits are well established in psychology. Persons with high scores in extraversion are more likely to be outgoing and enjoy social gatherings. Higher scores on agreeableness are related to being more trusting and helpful. Individuals who have higher scores in conscientiousness tend to plan out activities, be more organized, and methodical. Greater negative emotionality is related to experiencing more negative emotions such as anger or guilt. Those who show more open-mindedness tend to be curious and are open to trying new things [61]. In validating the BFI-2-XS all questions loaded strongly onto their respective five components of personality. Internal reliability Cronbach’s Alpha coefficients ranged from 0.83 to 0.90 for the subdomains, establishing adequate reliability and validity [60].

### Statistical analysis

All statistical analyses were completed using R Studio version 4.2.1 [62]. Serial ordinary least squares regression models were utilized to examine each psychosocial outcome: loneliness, stress, and memory complaints, and each social emotional outcome: social isolation, anxiety, and depression, as outlined in Macià et al. [63]. In addition to this analysis technique being completed in the literature exploring loneliness, a similar approach has been used to examine personality and self-reported hearing aid outcomes [47].

**Fig 1:**
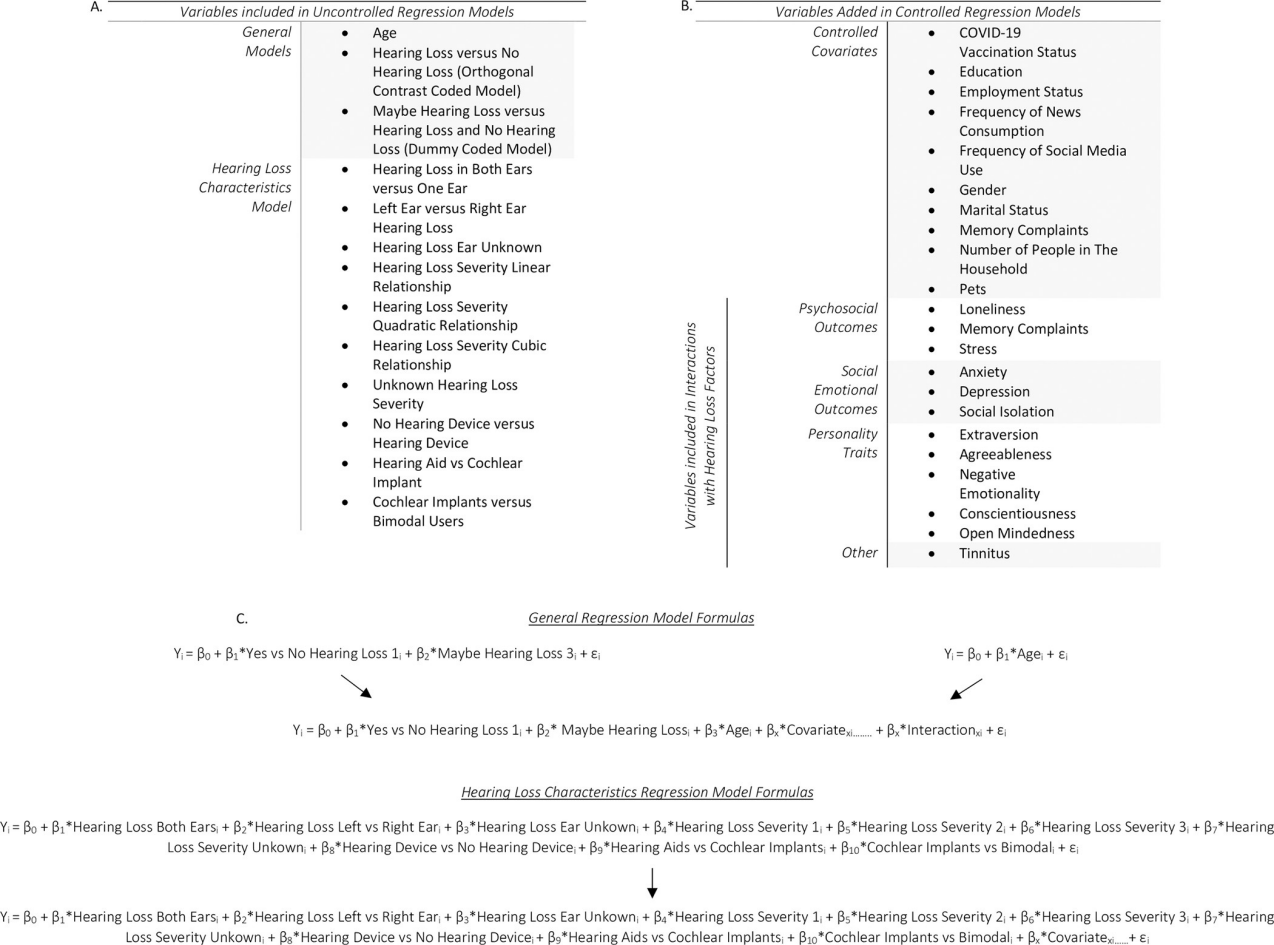
Regression models. This data was analyzed using a consecutive ordinary least squares regression approach. (A) First uncontrolled regression models were run with only a few variables. These included hearing loss only and age only models. The hearing loss only model was completed twice with orthogonal contrast codes and dummy codes to fully examine the differences across individuals who selected having hearing loss, maybe having hearing loss, and not having hearing loss. To further investigate hearing loss, a hearing loss characteristics model was completed, which divided hearing loss into several reported factors. (B) Additional demographic, psychosocial, social emotional, and personality trait variables were added to the controlled regression. Interaction terms were included between hearing loss variables and age, psychosocial, social emotional, personality traits, and tinnitus. (C) The age and hearing loss only regression utilized the same controlled regression for comparison. This model was completed twice to account for the different hearing loss codes. The controlled regression for the hearing loss characteristics model also included these covariates and interactions, but exchanged the hearing loss variable itself with the numerous hearing loss factors of interest. In these model formulas Y_i_ is the outcome variable of interest, β_0_ refers to the mean, and β_1-x_ correspond to the slope of each additional variable. β_x_ *Covariate_xi……_ implies the addition of all covariates listed in B. to the linear regression. β_x_ *Interaction_xi……_ implies the addition of all moderation terms with hearing loss factors listed in B. to the linear regression.

Therefore, regressions were first completed without controlling for any other factors for both hearing loss and age (see uncontrolled regression models Fig 1A). In the uncontrolled hearing loss models, hearing loss factors included comparisons for self-reported hearing loss versus no hearing loss, self-report of maybe having hearing loss in comparison to the other hearing ability options (no hearing loss and hearing loss), hearing loss versus maybe having hearing loss, and no hearing loss versus maybe having hearing loss. The hearing loss models were completed twice once with orthogonal contrast codes and dummy codes to fully examine the differences across individuals who selected having hearing loss, maybe having hearing loss and not having hearing loss.

Next a regression controlling for multiple possible covariates, as shown in Fig 1: General Models, were completed, and compared to the unadjusted regressions. In the controlled regression models, interaction variables were included for all hearing loss variables with age, psychosocial outcomes, social emotional outcomes, and personality traits. Furthermore, due to the high co-occurrence of tinnitus and hearing loss interaction variables between the presence of tinnitus and hearing loss variables were also included. Given that the variables of interest for this study were the moderation effects, main effects for psychosocial outcomes, social emotional outcomes, personality traits, and tinnitus are only discussed in relation to significant interactions but are reported in the tables in the results sections. In this larger regression, the employment options of retirement and disabled were combined due to a perfect correlation between these two categories.

To address the question of hearing loss characteristics the same method was utilized with a non-controlled and controlled regression for each outcome, which can be seen in Fig 1: Hearing Loss Characteristics. In these analyses, duration of device usage was not included due to the occurrence of multicollinearity between this variable and device type. Additionally, when examining memory complaints, a subset analysis was conducted in the same manner with participants aged 55 years or older due to the high incidence of memory concerns in this population [64].

Furthermore, controlling contrast coded variables with a Variance Inflation Factor (VIF) greater than five were removed from analysis, which included two contrast codes accounting for education and two contrast codes representing employment status. Histograms, Q-Q plots, Residuals vs Leverage plots, Scale-Location plots, and Residuals vs Fitted plots were first computed to assess potential violations in assumptions of normality and homogeneity of variance for all variables. Although variables with high VIF values were excluded, Residuals vs Fitted plots continued to demonstrate a patterned response. Visual inspection and outlier analyses were also performed.

P-values were adjusted to account for multiple comparisons using the Benjamini–Hochberg False Discovery Rate procedure to reduce the risk of type I errors [65]. Adjusted p-values are reported with an alpha of ≤0.05 utilized to signify statistical significance.

## Results

### Participant characteristics

One thousand and ninety-seven participants accessed the survey. Of these subjects 0.91% decided not to participate in the study, 9% did not correctly complete the verification questions within the survey, 18% had only partial survey responses, which resulted in a total sample of 891 participants utilized in the analysis of this survey. Participants were able to finish the survey over multiple days with one participant completing the survey in four days; however, among those who completed the survey in one day the average response time was 12 minutes. Using a power analysis with power (1 - β) set at 0.80 and α equal to 0.05 (two-tailed) indicated that 219 participants would be sufficient to detect a small effect size (d = 0.2). The mean age of participants in this study was approximately 50 years of age with a standard deviation of 18 years. Females accounted for the majority of respondents (76%), with the remaining sample being approximately 24% male and 0.78% identifying as other. Over half of the respondents reported having a college degree with 42.76% of the participants having a graduate degree, 31.51% of the participants having a bachelor’s degree, and 6.46% having an associate degree. Furthermore, 0.56% of the sample did not complete high school, 4.34% had a high school diploma, 2.12% received a technical degree, and 12.25% completed some college coursework. 53.25% of respondents indicated they were married not separated, while 46.75% of participants were not married. Two hundred and sixty-seven (30%) respondents self-reported having a hearing loss and 123 (14%) individuals indicated that they may have a hearing loss. For those with hearing loss, moderate hearing loss was most often indicated (41%), followed by severe hearing loss (34%), mild hearing loss (23%), and 2% of the 267 respondents with self-reported hearing loss did not know the severity of their hearing loss. Additionally, 22 CI users and 139 hearing aid users completed this survey. One hundred and fourteen individuals in this survey with self-reported hearing loss did not utilize a hearing device.

### Loneliness

Hearing loss was not associated with greater loneliness (F(1, 889) = 6.89, CI [0.05, 0.33], p = 0.097) in an uncontrolled model. In terms of age, a younger age was correlated with greater loneliness (F(1,890) = 35.61, CI [-0.03,-0.01], p<0.001) without the inclusion of hearing loss in the model.

When adding covariates and additional moderators (as noted in Fig 1B) to the model, hearing loss significantly interacted with another psychosocial measure. As demonstrated in Table 1, although there were no main effects of anxiety and hearing loss on loneliness, a significant additive interaction was found between hearing loss and anxiety (F(1,829) = 8.63, CI [0.06, 0.30], p = 0.044), to this extent having hearing loss and anxiety increased loneliness levels in comparison to people without hearing loss.

**Table 1:** Predictors effects on loneliness in a controlled model.

	Loneliness		
*Predictors*	*Estimates*	*CI*	*p*
**Hearing Loss**			
Yes vs. No Hearing Loss	0.19	0.05–0.33	0.102
Yes and No vs. Maybe Hearing Loss	0.04	-0.07–0.14	0.788
Yes vs. Maybe Hearing Loss	0.18	-0.18–0.53	0.672
No vs. Maybe Hearing Loss	-0.14	-0.49–0.21	0.748
**Age**			
Age	0.00	-0.01–0.01	0.991
**Tinnitus**			
Yes vs No Tinnitus	0.12	-0.03–0.27	0.454
Occasional Tinnitus	0.06	-0.03–0.14	0.589
**Personality Traits**			
Extraversion	-0.01	-0.06–0.03	0.837
Agreeableness	-0.01	-0.07–0.05	0.908
Negative Emotionality	0.12	0.06–0.17	**0.001** *
Conscientiousness	0.04	-0.01–0.09	0.520
Open Mindedness	0.07	0.01–0.12	0.128
**Psychosocial Outcomes**			
Memory Complaints	0.14	0.06–0.22	**0.018** *
Stress	0.11	0.05–0.16	**0.004** *
**Social Emotional Outcomes**			
Social Isolation	-0.07	-0.10 –-0.05	**<0.001** *
Anxiety	0.00	-0.14–0.13	0.990
Depression	0.13	0.01–0.25	0.257
**Significant Interactions**			
Yes vs. No Hearing Loss * Anxiety	0.18	0.06–0.30	**0.044** *

Loneliness was measured using the Three-Item Loneliness Scale, in which higher scores are indicative of greater loneliness. Estimated coefficients for categorical variables (hearing loss and tinnitus outcomes) denote the difference in average ratings between the groups (i.e., hearing loss vs no hearing loss, tinnitus vs no tinnitus, etc.). For continuous measures (personality traits, psychosocial outcomes, and social emotional outcomes), the coefficients indicate the predicted change in the outcome variable for each 1 unit increase in the predictor variable. While interaction terms included all predictor variables with hearing loss outcomes, only significant interactions are included in this table. These interactions signify that hearing loss outcomes vary the effects of other predictor variables on loneliness. In this controlled linear regression, hearing loss was not associated with loneliness, but interacted significantly with anxiety to produce an additive effect of hearing loss and anxiety on increases in loneliness.

*p ≤ 0.05

In examining specific hearing loss characteristics in an uncontrolled model, loneliness increased as a function of severity of hearing loss (F(1, 878) = 10.89, CI [0.24, 0.96], p = 0.018), such that as hearing loss severity increased reported loneliness scores increased as well. There was a trend such that, not having a hearing device was associated with greater loneliness scores (F(1, 878) = 8.34, CI [0.10, 0.54], p = 0.053) in comparison to those with hearing aids and CIs. Additionally, there was a significant difference in loneliness between those who reported utilizing a hearing aid and a CI (F(1,878) = 9.92, CI [0.26, 1.11], p = 0.030), such that hearing aid users on average scored 0.68 points higher on loneliness. There was a significant negative interaction between hearing loss severity and using a hearing aid, as well (F(1,878) = 13.89, CI [-0.55, -0.17], p = 0.005). This suggests that as hearing loss severity increased there was less of a difference in reported loneliness between hearing aid users and CI users.

In the hearing loss characteristics model after the addition of the covariates only the negative interaction between hearing loss severity and hearing aid use remained significant (F(1,818) = 12.97, CI [-0.44, -0.13], p = 0.007), while there was a trend for increases in hearing loss severity being associated with increases in loneliness scores (F(1,818) = 8.21, CI [0.13, 0.72], p = 0.053).

### Social isolation

Surprisingly there was no significant effect of hearing loss on social isolation (F(1, 889) = 4.41, CI [-0.85, -0.03], p = 0.252) in an uncontrolled model. However, there was a trend towards a significant difference between maybe having hearing loss and not having hearing loss, such that those who selected maybe having hearing loss indicated less social engagement (F(1, 889) = 7.34, CI [0.41, 2.58], p = 0.078). Advancing age was negatively correlated with social engagement when not taking hearing loss or other covariates into account, such that older individuals reported higher levels of social isolation (F(1,890) = 10.28, CI [-0.05, -0.01], p = 0.018).

Table 2 demonstrates that upon adding covariates and additional moderator variables (see Fig 1B), age remained a significant predictor of social isolation (F(1, 829) = 23.21, CI [-0.11, -0.05], p<0.001), such that older age was associated with more social isolation. There was a trend towards an interaction between maybe having hearing loss and stress (F(1, 829) = 7.43, CI [-1.22, -0.20], p = 0.079). As stress levels increased, maybe having hearing loss resulted in greater social isolation than that exhibited by those self-identifying as having hearing loss.

**Table 2:** Predictors effects on social isolation in a controlled model.

	Social Isolation		
*Predictors*	*Estimates*	*CI*	*p*
**Hearing Loss**			
Yes vs. No Hearing Loss	0.20	-0.27 – 0.68	0.730
Yes and No vs. Maybe Hearing Loss	-0.09	-0.43 – 0.24	0.839
Yes vs. Maybe Hearing Loss	0.66	-0.49 – 1.82	0.660
No vs. Maybe Hearing Loss	0.41	-0.72 – 1.55	0.773
**Age**			
Age	-0.08	-0.11 – -0.05	**<0.001** *
**Tinnitus**			
Yes vs No Tinnitus	-0.37	-0.86 – 0.12	0.502
Occasional Tinnitus	-0.09	-0.38 – 0.19	0.802
**Personality Traits**			
Extraversion	0.42	0.26 – 0.58	**<0.001** *
Agreeableness	0.20	0.02 – 0.39	0.244
Negative Emotionality	0.05	-0.14 – 0.23	0.862
Conscientiousness	-0.07	-0.24 – 0.09	0.711
Open Mindedness	0.22	0.05 – 0.40	0.124
**Psychosocial Outcomes**			
Loneliness	-0.77	-1.02 – -0.53	**<0.001** *
Memory Complaints	0.10	-0.17 – 0.36	0.774
**Stress**			
Social Emotional Outcomes	-0.09	-0.28 – 0.09	0.672
Anxiety	0.07	-0.38 – 0.51	0.927
Depression	-0.47	-0.87 – -0.07	0.167
**Significant Interactions** *			
Treated Hearing Loss vs. Maybe Hearing Loss * Stress	-0.71	-1.22 – -0.20	0.079^+^

Social isolation was measured using the LSNS-6, in which lower scores are indicative of greater social isolation. Estimated coefficients for categorical variables (hearing loss and tinnitus outcomes) denote the difference in average ratings between the groups (i.e., hearing loss vs no hearing loss, tinnitus vs no tinnitus, etc.). For continuous measures (personality traits, psychosocial outcomes, and social emotional outcomes), the coefficients indicate the predicted change in the outcome variable for each 1 unit increase in the predictor variable. While all other predictor variables were included in interactions with hearing loss outcomes, only significant interactions are included in this table. While hearing loss was not associated with social isolation, identifying as maybe having hearing loss showed a trend towards interacting with increasing stress to produce an additive effect of greater social isolation than hearing loss peers.

*p < 0.05

+p < 0.08

In the controlled hearing loss characteristics model, as extraversion increased, social engagement was predicted to significantly increase (F(1, 818) = 49.80, CI [0.32, 0.57], p<0.001), with a trend towards a significant negative interaction between hearing loss severity and extraversion (F(1, 818) = 7.92, CI [-0.27, -0.05], p = 0.062).This trend results in extraversion no longer resulting in greater social engagement above a mild degree of hearing loss.

### Stress

In the overall uncontrolled and controlled hearing loss and hearing loss characteristics models, hearing loss was not a significant predictor of stress nor were there significant interactions with hearing loss on stress (see Table 3).

**Table 3:** Predictors effects on stress in a controlled model.

	Perceived Stress		
*Predictors*	*Estimates*	*CI*	*p*
**Hearing Loss**			
Yes vs. No Hearing Loss	-0.16	-0.37–0.04	0.469
Yes and No vs. Maybe Hearing Loss	-0.02	-0.16–0.13	0.957
Yes vs. Maybe Hearing Loss	-0.17	-0.67–0.34	0.794
No vs. Maybe Hearing Loss	0.12	-0.37–0.62	0.862
**Age**			
Age	-0.01	-0.02–0.01	0.720
**Tinnitus**			
Yes vs No Tinnitus	0.14	-0.08–0.35	0.596
Occasional Tinnitus	-0.15	-0.28 –-0.03	0.128
**Personality Traits**			
Extraversion	-0.01	-0.08–0.05	0.898
Agreeableness	-0.03	-0.11–0.05	0.755
Negative Emotionality	0.14	0.07–0.22	0.007*
Conscientiousness	-0.12	-0.19 –-0.05	0.018*
Open Mindedness	0.01	-0.07–0.08	0.953
**Psychosocial Outcomes**			
Loneliness	0.20	0.10–0.31	0.005*
Memory Complaints	0.23	0.12–0.34	0.002*
**Social Emotional Outcomes**			
Social Isolation	-0.02	-0.05–0.02	0.664
Anxiety	0.66	0.48–0.84	<0.001*
Depression	0.65	0.48–0.81	<0.001*

Stress was measured using the PSS-4, in which higher scores are indicative of more stress. Estimated coefficients for categorical variables (hearing loss and tinnitus outcomes) denote the difference in average ratings between the groups (i.e., hearing loss vs no hearing loss, tinnitus vs no tinnitus, etc.). For continuous measures (personality traits, psychosocial outcomes, and social emotional outcomes), the coefficients indicate the predicted change in the outcome variable for each 1 unit increase in the predictor variable. While all other predictor variables were included in interactions with hearing loss outcomes, no significant interactions were found and therefore are not listed in the table.

*p < 0.05

In a regression model only including age, age was negatively correlated with stress (F(1,890) = 133.63, CI [-0.08, -0.06], p<0.001), consequently younger respondents reported greater stress on average. In the controlled regression, there was no longer a significant relationship between age and stress (F(1, 829) = 0.741, CI [-0.02, 0.01], p = 0.720).

### Anxiety

There was a trend towards a relationship between anxiety and hearing loss (F(1,889) = 8.15, CI [-0.27, -0.05], p = 0.053), such that those without hearing loss reported higher anxiety levels on average in an uncontrolled regression model. Age was negatively correlated with increasing anxiety levels (F(1,890) = 141.13, CI [-0.03, -0.02], p<0.001) in an uncontrolled model, and was trending towards a negative correlation with anxiety in the larger model (F(1,829) = 7.91, CI [-0.02, 0.00], p = 0.062), when covariates and additional moderating variables (see Fig 1B) were added to the model.

As shown in Table 4, when covariates were added to the model, there was a significant additive relationship between loneliness and hearing loss (F(1,829) = 9.61, CI[0.03, 0.13], p = 0.030). It is important to note that this significant interaction was also noted above when examining loneliness as an outcome measure.

**Table 4:** Predictors effects on anxiety in a controlled model.

	Anxiety		
*Predictors*	*Estimates*	*CI*	*p*
**Hearing Loss**			
Yes vs. No Hearing Loss	0.02	-0.08 – 0.11	0.910
Yes and No vs. Maybe Hearing Loss	-0.03	-0.10 – 0.04	0.757
Yes vs. Maybe Hearing Loss	0.13	-0.11 – 0.37	0.664
No vs. Maybe Hearing Loss	0.12	-0.11 – 0.36	0.666
**Age**			
Age	-0.01	-0.02 – -0.00	0.062
**Tinnitus**			
Yes vs No Tinnitus	0.00	-0.11 – 0.10	0.978
Occasional Tinnitus	0.00	-0.06 – 0.06	0.991
**Personality Traits**			
Extraversion	0.04	0.01 – 0.07	0.102
Agreeableness	0.04	0.00 – 0.07	0.353
Negative Emotionality	0.16	0.13 – 0.20	<0.001*
Conscientiousness	0.03	-0.01 – 0.06	0.502
Open Mindedness	-0.01	-0.04 – 0.03	0.910
**Psychosocial Outcomes**			
Loneliness	-0.03	-0.08 – 0.03	0.674
Memory Complaints	0.02	-0.03 – 0.08	0.738
Stress	0.13	0.09 – 0.17	<0.001*
**Social Emotional Outcomes**			
Social Isolation	0.00	-0.02 – 0.02	0.982
Depression	0.18	0.10 – 0.27	<0.001*
**Significant Interactions**			
Yes vs. No Hearing Loss * Loneliness	0.08	0.03 – 0.13	0.030*

Anxiety was measured using the PHQ-4, in which higher scores are indicative of greater anxiety. Estimated coefficients for categorical variables (hearing loss and tinnitus outcomes) denote the difference in average ratings between the groups (i.e., hearing loss vs no hearing loss, tinnitus vs no tinnitus, etc.). For continuous measures (personality traits, psychosocial outcomes, and social emotional outcomes), the coefficients indicate the predicted change in the outcome variable for each 1 unit increase in the predictor variable. While all other predictor variables were included in interactions with hearing loss outcomes, only significant interactions are included in this table. Similar to the findings when loneliness was the outcome measure, there was a significant additive interaction on the effect of hearing loss on anxiety with increasing levels of loneliness without a main effect of hearing loss on anxiety.

*p ≤ 0.05

The uncontrolled and controlled hearing loss characteristics model overall were not significantly related to anxiety scores.

### Depression

Depression was negatively correlated with age (F(1,890) = 60.09, CI [-0.02, -0.01], p<0.001) when not controlling for any covariates. No relationship between hearing loss and depression (F(2,889) = 0.91, p = 0.730) or hearing loss characteristics and depression (F(13,878) = 0.58, p = 0.966) were noted in the omnibus uncontrolled regressions (see Table 5).

**Table 5:** Predictors effects on depression in a controlled model.

	Depression		
*Predictors*	*Estimates*	*CI*	*p*
**Hearing Loss**			
Yes vs. No Hearing Loss	-0.04	-0.14 – 0.05	0.724
Yes and No vs. Maybe Hearing Loss	0.04	-0.03 – 0.11	0.664
Yes vs. Maybe Hearing Loss	-0.15	-0.38 – 0.09	0.599
No vs. Maybe Hearing Loss	-0.05	-0.28 – 0.18	0.896
**Age**			
Age	0.00	-0.01 – 0.01	0.924
**Tinnitus**			
Yes vs No Tinnitus	0.01	-0.09 – 0.11	0.967
Occasional Tinnitus	0.02	-0.03 – 0.08	0.755
**Personality Traits**			
Extraversion	-0.02	-0.05 – 0.01	0.599
Agreeableness	0.02	-0.02 – 0.06	0.664
Negative Emotionality	0.06	0.02 – 0.10	**0.030** *
Conscientiousness	-0.03	-0.06 – 0.01	0.438
Open Mindedness	0.04	0.00 – 0.07	0.300
**Psychosocial Outcomes**			
Loneliness	0.07	0.02 – 0.12	0.079+
Memory Complaints	0.07	0.01 – 0.12	0.128
Stress	0.14	0.10 – 0.17	**<0.001** *
**Social Emotional Outcomes**			
Social Isolation	-0.02	-0.03 – 0.00	0.262
Anxiety	0.19	0.11 – 0.28	**<0.001** *

Depression was measured using the PHQ-4, in which higher scores are indicative of greater depressive symptoms. Estimated coefficients for categorical variables (hearing loss and tinnitus outcomes) denote the difference in average ratings between the groups (i.e., hearing loss vs no hearing loss, tinnitus vs no tinnitus, etc.). For continuous measures (personality traits, psychosocial outcomes, and social emotional outcomes), the coefficients indicate the predicted change in the outcome variable for each 1 unit increase in the predictor variable. In this controlled linear regression, hearing loss was not associated with depressive symptoms, and there were no significant interactions between hearing loss, personality traits, psychosocial outcomes, or social emotional outcomes.

*p ≤ 0.05

+p < 0.08

In the controlled regression model, age was no longer a significant predictor of depression (F(1,829) = 0.09, CI [-0.01, 0.01], p = 0.924). In the controlled hearing loss characteristics regression, there was a trend such that as hearing loss severity increased depression scores increased indicating the presence of a linear relationship (F(1,818) = 8.43, CI [-0.49, -0.09], p = 0.053). There was a trend towards an interaction between hearing loss severity and not having a hearing device (F(1,818) = 7.94, CI [0.03, 0.14],p = 0.062), indicating that at higher levels of hearing loss severity individuals without a hearing device had higher depression scores.

### Memory complaints

In our overall uncontrolled regression model, age was not related to memory complaints as measured by the SCF questions (F(1,890) = 3.12, p = 0.390). However, hearing loss in an uncontrolled model was associated with greater memory complaints (F(1,889) = 18.05, CI [0.14, 0.38], p<0.001).

Surprisingly in the controlled regression model, as demonstrated in Table 6, age remained unrelated to memory complaints (F(1,829) = 3.01, CI [0.00, 0.02], p = 0.540). There was a trending relationship between hearing loss and maybe having hearing loss interacting with negative emotionality (F(1,829) = 8.38, CI [0.07, 0.37], p = 0.053). At higher levels of negative emotionality, having hearing loss resulted in more memory complaints than those who maybe have a hearing loss.

**Table 6:** Predictors effects on memory complaints in a controlled model.

	Memory Complaints			
*Predictors*	*Estimates*	*CI*		*p*
**Hearing Loss**				
Yes vs. No Hearing Loss	0.17	0.02 – 0.31		0.182
Yes and No vs. Maybe Hearing Loss	-0.03	-0.13 – 0.07		0.802
Yes vs. Maybe Hearing Loss	0.28	-0.07 – 0.63		0.469
No vs. Maybe Hearing Loss	-0.06	-0.40 – 0.28		0.910
**Age**				
Age	0.01	0.00 – 0.02		0.540
**Tinnitus**				
Yes vs No Tinnitus	0.10	-0.05 – 0.25		0.558
Occasional Tinnitus	-0.04	-0.13 – 0.04		0.669
**Personality Traits**				
Extraversion	0.01	-0.04 – 0.05		0.928
Agreeableness	0.01	-0.05 – 0.07		0.914
Negative Emotionality	-0.02	-0.08 – 0.03		0.714
Conscientiousness	-0.11	-0.16 – -0.06		**<0.001** *
Open Mindedness	-0.04	-0.09 – 0.02		0.550
**Psychosocial Outcomes**				
Loneliness	0.12	0.04 – 0.19		**0.030** *
Stress	0.11	0.05 – 0.16		**0.002** *
**Social Emotional Outcomes**				
Social Isolation	0.01	-0.01 – 0.04		0.699
Anxiety	0.07	-0.06 – 0.20		0.669
**Significant Interactions** *				
Yes vs. Maybe Hearing Loss * Negative Emotionality	0.22	0.07 – 0.37		0.053^+^

Memory complaints were measured using the SCF, in which higher scores are indicative of greater recent memory concerns. Estimated coefficients for categorical variables (hearing loss and tinnitus outcomes) denote the difference in average ratings between the groups (i.e., hearing loss vs no hearing loss, tinnitus vs no tinnitus, etc.). For continuous measures (personality traits, psychosocial outcomes, and social emotional outcomes), the coefficients indicate the predicted change in the outcome variable for each 1 unit increase in the predictor variable. While all other predictor variables were included in interactions with hearing loss outcomes, only significant interactions are included in this table. In this controlled linear regression, hearing loss was not associated with memory complaints, although there was a significant positive relationship between memory complaints and hearing loss in the uncontrolled linear regression. There was a trend towards negative emotionality interacting with hearing loss such that there were greater memory complaints among those with hearing loss in comparison to those who indicated maybe having hearing loss in those individuals with greater negative emotionality.

*p ≤ 0.05

+p < 0.08

In a deeper analysis of hearing loss characteristics in an uncontrolled model, there was a trend that respondents who indicated that the ear with hearing loss was unknown were more likely to report memory difficulties via their responses to the SCF questions (F(1, 878) = 8.50, CI [0.14, 0.73], p = 0.053). In the controlled hearing loss characteristics model, not knowing which ear had hearing loss was associated with more memory difficulties (F(1,818) = 11.40, CI [0.20, 0.74], p = 0.018). There was a trend such that not using a hearing device interacted with depression (F(1,818) = 7.97, CI [-0.23, -0.04], p = 0.062). At higher levels of depression, having a hearing device was associated with increased memory complaints in comparison to not having a hearing device.

The same analyses as described above were also completed in a subset of respondents aged 55 years or older. In the uncontrolled models, hearing loss was still associated with greater memory concerns (F(1, 394) = 21.31, CI[0.22, 0.55], p<0.001). Age continued to not demonstrate a relationship with memory complaints (F(1, 395) = 4.45, CI[-0.04, 0.00], p = 0.250). In the controlled model, there was a trend for greater memory concerns to be reported in those with hearing loss (F(1,335) = 727, CI[0.08, 0.52], p = 0.079).

In terms of hearing loss characteristics, there was a trend that having hearing loss and no device was associated with greater memory complaints (F(1, 383) = 8.31, CI[0.10, 0.55], p = 0.053). Additionally there was a trend, such that having a hearing aid was associated with greater memory complaints than having a CI (F(1, 383) = 7.90, CI[0.18, 1.03], p = 0.062). However, when covariates were added to the models, there were no significant relationships between hearing loss, age, or hearing loss characteristics nor any significant interactions with these variables of interest.

## Discussion

The results from this study aid in the understanding of the complex relationship between hearing loss, psychosocial outcomes, and personality traits. This study found that increases in memory complaints were associated with hearing loss. Hearing loss was positively correlated with recent difficulty with memory-based tasks, as measured by the SCF questions, when examining all adults and older adults (>54 years of age) as has been noted in previous studies [54, 55, 66–69]. However, the addition of covariates (Fig 1B) and additional predictors (personality traits, psychosocial outcomes, and social emotional outcomes) resulted in hearing loss no longer being associated with increased memory complaints. A recent study by Lin et al. [70] found differences in rates of cognitive decline were affected by hearing aids only in a subpopulation of individuals with hearing loss and cardiac risk factors. Taken together with our findings, it may suggest that the link between hearing loss, hearing treatments, and cognition is only present or stronger in certain individuals [70]. Future research is needed in this area to determine which individual or personality characteristics may place hearing patients at increased risk for cognitive decline. Furthermore, researchers would benefit from carefully considering possible covarying factors when examining hearing loss and cognition [71]. For instance, while this study did not find hearing loss to remain a significant predictor of memory complaints when controlling for many other factors such as demographics, social emotional outcomes and personality traits, our other psychosocial outcomes (stress and loneliness) showed a significant positive relationship between stress and loneliness to memory concerns. This may suggest that caution should be taken when examining cognition to also account for other psychosocial outcomes.

Consistent with Dawes et al. [9], the results of this study did not find significant interactions between social isolation and depression with hearing loss on the effects of cognition. Dawes et al. [9] also examined if the relationship between cognition and hearing was mediated by social isolation and depression and noted it was unlikely that the effect of hearing and hearing treatment on cognition is mediated by these factors. However, personality traits did interact with the effects of hearing loss on memory concerns. Previous research has revealed a link between higher negative emotionality with greater cognitive decline [39, 40]. Our results further demonstrate this, in that, having a more negative personality and hearing loss rather than ‘maybe’ having hearing loss created an additive effect in increasing reports of memory complaints.

Furthermore, this study demonstrated that the presence and severity of hearing loss can have differential effects on known relationships between personality and psychosocial outcomes. Interestingly, greater hearing loss negated the relationship between extraversion and social engagement. These outcomes may be seen as complementary to Stephan et al.’s [42] findings of greater rates of decline in extraversion for individuals with hearing loss. Further, Cox et al. [47] noted that higher neuroticism and lower extraversion were correlated with greater activity limitations and participation restrictions in people obtaining hearing aids. These significant interactions suggest that it may be useful to consider personality traits in those with hearing loss when planning intervention programs. For instance, it may be that our extraverted patients need more support as their social engagement and levels of loneliness could be more affected than introverted patients.

In the controlled regression models, which added numerous variables (see Fig 1B), hearing loss significantly interacted with other outcome factors such as anxiety, loneliness, and stress. The presence of anxiety and hearing loss had an additive effect on increased loneliness, while the presence of loneliness and hearing loss had an additive effect on increased anxiety. This three-way interaction was present regardless of the outcome measure of interest (i.e., loneliness or anxiety) suggesting this is a robust finding. Previous studies have shown increases in anxiety with increases in hearing loss severity [12, 14, 72]. In examining the relationship between stress and social isolation it was found that as social isolation increased, stress increased with those who selected ‘maybe’ having hearing loss showing the greatest amount of stress linked to social isolation. Previous research has found high levels of social loneliness in individuals who self-report hearing loss, and it has been noted that those with less acceptance of their hearing loss and more stress reported greater social loneliness [73]. Given that we separately examined those who indicated maybe having hearing loss, our findings could be reflecting a lack of acceptance of hearing loss interacting with stress. Furthermore, Jayakody et al. [14] found that even with only high frequency hearing loss at 6000 and 8000 Hz there was an increased risk of anxiety and stress. These respondents, who often would not be considered clinically for hearing treatments, may also be the same individuals that would self-identify as maybe having hearing loss. These findings suggest that future research should examine the effects of loneliness and social isolation in the ‘maybe’ hearing loss population separate from the non-hearing loss and hearing loss populations.

Overall, the results from this study replicate results from previous studies on hearing loss and personality relationships [42, 47] as well as hearing loss and wellbeing [9, 12, 14, 39, 40, 72]. However, this study expands on those findings by including both relationships of personality and wellbeing and their interaction with hearing loss. Given that this study found interactions between personality and memory outcomes, future research may wish to explore if targeted interventions could improve memory benefits obtained from hearing loss treatments. Furthermore, research could explore if audiological rehabilitation programs are more effective for certain personality traits. For instance, social support groups may be particularly effective for those with higher levels of extroversion given that this study found greater decreases in social engagement in these individuals with increasing levels of hearing loss. Lastly, findings in this study indicate that that those who think they maybe have a hearing loss have differences in their wellbeing between those with and without hearing loss. This may be a population of interest for future researchers as over-the-counter hearing aids are intended for perceived hearing loss rather than diagnosed hearing loss.

It is important to note that while the above findings are compelling, there were limitations with the study. The results of this study are correlational and therefore causation cannot be determined. This study utilized validated questionnaires; however, many of these questionnaires employ Likert-scales, which provide limited choice possibilities and are prone to acquiescence bias [74]. Furthermore, the short form versions of the anxiety and depression screener may have resulted in floor effects being obtained on these measures. This study was completed with a convenience sample, therefore results on some of the outcomes did not vary greatly between respondents. Those individuals who choose to participate in survey research when convenience samples are utilized may differ from those who choose not to partake in research [75]. Future studies should attempt a random sampling approach to better reflect the general population.

The current study focused on self-reported performance on memory-based tasks as a measure of Subjective Cognitive Function. In a large longitudinal data set of 454 participants, it was found that higher responses to the SCF questions at baseline were related to increased odds of progression to mild cognitive impairment measured using an objective neurocognitive test battery. The authors suggest that the presence of subjective cognitive complaints prior to a measurable mild cognitive impairment diagnosis may represent a transitional phase to mild cognitive impairment [76]. While our methodology focused on memory, a method employed by others to evaluate cognition and cognitive decline [53–55, 76], it is important to recognize that cognition also involves attention, sensory processing, perception, and processes that regulate, retain, manipulate, and integrate information [77]. As previous studies have done, the current study focused on memory as our cognitive aspect of interest, rather than other aspects of cognition. Given our interest in potential cognitive changes associated with hearing loss, an emphasis on memory, which is relatively easy to evaluate via self-report, is justified. Given the design of the current study, self-reported measures of perception, attention, sensation, and other cognitive processes (such as the ability to integrate information) would be more difficult for subjects to self-evaluate and report accurately, increasing the risk of self-reporting errors. Future studies that employ direct measures of cognition and hearing should consider including additional measures of other aspects cognition than were used here.

Self-report bias is another caution of this study. While some research has demonstrated that self-reported hearing loss methods are sensitive to objective measures of hearing loss thresholds [48–50], self-reported hearing loss has also been shown to result in many people under or over reporting their hearing loss [78]. Specifically, younger respondents tend to overestimate while older respondents underestimate their hearing loss [78]. Responses to the SCF questions are also self-reported evaluations of memory/cognition and are also vulnerable to either over or underestimation.

Finally, this study was conducted during the COVID-19 pandemic, which may have impacted our results. In examining those with hearing loss, Dunn et al. [22] found less social isolation and anxiety in CI users during the pandemic. In surveys examining the general population, high stress was reported by participants [79, 80]. These studies demonstrate that that the COVID-19 pandemic may have differentially impacted the hearing loss and non-hearing loss populations. Furthermore, a recent review suggests that in older individuals, feelings of loneliness increased during the pandemic [81]. Shrira et al. [82] also noted that the link between feelings of loneliness and mental health outcomes during the COVID-19 pandemic was stronger in those who perceived themselves as being older. Additionally, in a meta-analysis examining the link between prolonged feelings of loneliness and social isolation, it was shown that older adults experiencing these were at greater risk for developing dementia [83]. Since this study included older adults (mean age = 50 years) and examined psychosocial outcomes that could affect each other as well as have been affected by the COVID-19 pandemic, the results reported from this study could have been inflated by the COVID-19 pandemic. Research on this topic should continue and be extended beyond the COVID-19 pandemic to fully understand the impact of COVID-19 on these results.

## Conclusion

To summarize the main findings of our study, hearing loss is related in a complex fashion to many psychosocial outcomes and social emotional factors. In addition to the known relationships between hearing loss, loneliness, and cognition, our study demonstrates that other social emotional factors such as anxiety, and stress can strengthen these relationships as a function of hearing loss. These interactions were different for those who thought they may have hearing loss in comparison to those with hearing loss or without hearing loss. Furthermore, our study suggests that hearing loss interacts with personality in complex ways. For example, individuals with greater negative emotionality and hearing loss tended to report more memory complaints. The association between hearing loss and memory complaints was also found to change as a function of controlling variables, such as controlling for social emotional and psychosocial outcomes. In reviewing the associations between hearing loss, cognitive impairment, depression, anxiety, and quality of life, Blazer & Tucci [84] noted that mental health professionals required a greater knowledge of hearing loss to allow for enhanced psychotherapies in this population. Our results provide useful information for mental health professionals and hearing health care professionals who treat patients with hearing loss regarding psychosocial and social emotional influences of hearing loss, and how personality may shape these relationships. A deeper insight into these relationships may produce better treatment outcomes and allow for customizable aural rehabilitation programs.

## Supporting information

S1 Appendix Study questionnaire. All questionnaires (Three-Item Loneliness, PSS-4, SCF, LSNS-6, PHQ-4, BFI-2-XS) utilized in this study are included. In addition, all demographic information collected from respondents are presented.
(DOCX)

S2 Appendix Study data. (CSV)
