# Supplementary material for: Hearing loss and psychosocial outcomes: Influences of social emotional aspects and personality
Source: PLoS One. 2024 Jun 12;19(6):e0304428. doi: 10.1371/journal.pone.0304428 (PMC11168651; doi:10.1371/journal.pone.0304428)
Supplement: S1 Appendix — All questionnaires (Three-Item Loneliness, PSS-4, SCF, LSNS-6, PHQ-4, BFI-2-XS) utilized in this study are included. In addition, all demographic information collected from respondents are presented. (DOCX) [file pone.0304428.s001.docx]

Start of Block: Demographics

Q2 How old are you?

________________________________________________________________

Skip To: End of Survey If Condition: How old are you? Is Less Than or Equal to 17. Skip To: End of Survey.

Q5 In which state do you currently reside?

▼ Alabama ... I do not reside in the United States

Skip To: End of Survey If 50 States, D.C. and Puerto Rico != I do not reside in the United States

Q3 I identify as

- Male
- Female
- Other

Q4 What is your level of education?

- Some High School Coursework Completed
- High School Diploma or Equivalent
- Technical Degree
- Some College Coursework Completed
- Associate's Degree
- Bachelor's Degree
- Master's/Doctoral Degree

Q7 Which of the following best describes your current residential area?

- Rural
- Urban
- Suburban

|  |
| --- |

Q8 Which of the following options most accurately describes your relationship status?  *Please* *select* *all* *that* *apply*.

- Married
- Widowed
- Divorced
- Separated
- Single
- Living with a Partner
- Living with a Roommate

Q9 How many people, including yourself, are currently living or staying at your home?

- 1
- 2
- 3
- 4
- 5
- 6
- More than 6

Q10 Do you have pet living in your home?

- Yes
- No

| 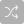 |
| --- |

Q11 Which statement best describes your current employment status?
*Please select all that apply.*

- Working (remotely)
- Working (in person)
- Working (self-employed)
- Not working (temporary layoff from a job)
- Not working (looking for work)
- Not working (retired)
- Not working (disabled)
- Not working (other) ________________________________________________
- Student (remote)
- Student (in person)
- Prefer not to answer

Q12 How much do you watch or listen to news media?

- A great deal
- A lot
- A moderate amount
- A little
- Not at all

Q13 How frequently do you use social media?

- A great deal
- A lot
- A moderate amount
- A little
- Not at all

Q14 Have you been fully vaccinated against COVID-19?

- Yes
- Maybe
- No

Q15 Do you experience noises or ringing in your ears that is bothersome on a regular basis?

- Yes
- Sometimes
- No

Q16 Do you have hearing loss?

- Yes
- Maybe
- No

Skip To: End of Block If Do you have hearing loss? = No

Skip To: End of Block If Do you have hearing loss? = Maybe

Q17 In which ear do you have a hearing loss?

- Left
- Right
- Both
- Unknown

Q18 What is the severity of your hearing loss?

- Mild
- Moderate
- Severe
- Unknown

Q19 Which of the following devices do you currently use? 
*Please select all that apply.*

- Hearing Aid
- Cochlear Implant
- None of the above

Q20 Approximately how long have you used hearing devices?

- < 1 year
- < 5 years
- > 5 years
- I have not used any hearing devices

End of Block: Demographics

Start of Block: BFI-2

Q21
Here are a number of characteristics that may or may not apply to you. Next to each statement please indicate the extent to which you agree or disagree with that statement.

|  | Disagree strongly | Disagree a little | Neutral: no opinion | Agree a little | Agree strongly |
| --- | --- | --- | --- | --- | --- |
| **I am someone who...** tends to be quiet |  |  |  |  |  |
| **I am someone who...** is compassionate, has a soft heart |  |  |  |  |  |
| **I am someone who...** tends to be disorganized |  |  |  |  |  |
| **I am someone who...** worries a lot |  |  |  |  |  |
| **I am someone who...** is fascinated by art, music, or literature |  |  |  |  |  |
| **I am someone who...** is dominant, acts as a leader |  |  |  |  |  |
| **I am someone who...** is sometimes rude to others |  |  |  |  |  |
| **For this question...**  please select agree strongly |  |  |  |  |  |
| **I am someone who...** has difficulty getting started on tasks |  |  |  |  |  |
| **I am someone who...** tends to feel blue |  |  |  |  |  |
| **I am someone who...** has little interest in abstract ideas |  |  |  |  |  |
| **I am someone who...** is full of energy |  |  |  |  |  |
| **I am someone who...** assumes the best about people |  |  |  |  |  |
| **I am someone who...** is reliable, can always be counted on |  |  |  |  |  |
| **I am someone who...** is emotionally stable, not easily upset |  |  |  |  |  |
| **I am someone who...** is original, comes up with new ideas |  |  |  |  |  |

End of Block: BFI-2

Start of Block: PSS

Q22 These questions ask you about your feelings and thoughts **during the last month.** In each case, you will be asked to indicate how often you felt or thought a certain way.

|  | Never | Almost never | Sometimes | Fairly often | Very often |
| --- | --- | --- | --- | --- | --- |
| In the last month, how often have you felt that you were unable to control the important things in your life? |  |  |  |  |  |
| In the last month, how often have you felt confident about your ability to handle your personal problems? |  |  |  |  |  |
| In the last month, how often have you felt that things were going your way? |  |  |  |  |  |
| In the last month, how often have you felt difficulties were piling up so high that you could not overcome them? |  |  |  |  |  |

End of Block: PSS

Start of Block: PHQ-4

Q23 The following questions will ask how often you have been bothered by certain problems in the **last two weeks**.

|  | Not at all | Several days | More than half the days | Nearly every day |
| --- | --- | --- | --- | --- |
| Over the last 2 weeks, how often have you been bothered by feeling nervous, anxious, or on edge? |  |  |  |  |
| Over the last 2 weeks, how often have you been bothered by not being able to stop or control worrying? |  |  |  |  |
| Over the last 2 weeks, how often have you been bothered by little interest or pleasure in doing things? |  |  |  |  |
| Over the last 2 weeks, how often have you been bothered by feeling down, depressed, or hopeless? |  |  |  |  |

End of Block: PHQ-4

Start of Block: Three-Item Loneliness Scale

Q24
The next questions are about how you feel about different aspects of your life. For each one, select how often you feel that way.

|  | Hardly ever | Some of the time | Often |
| --- | --- | --- | --- |
| First, how often do you feel that you lack companionship? |  |  |  |
| How often do you feel left out? |  |  |  |
| How often do you feel isolated from others? |  |  |  |
| How often do you feel happy spending time by yourself? |  |  |  |
| How often do you feel solitude affects you in a positive way? |  |  |  |

End of Block: Three-Item Loneliness Scale

Start of Block: Lubben Social Network Scale 1

Q25
The following questions ask about family:

|  | Never | One | Two | Three or Four | Five Thru Eight | Nine or More |
| --- | --- | --- | --- | --- | --- | --- |
| How many relatives do you see or hear from at least once a month? |  |  |  |  |  |  |
| How many relatives do you feel at ease with that you can talk about private matters? |  |  |  |  |  |  |
| Please select Two here |  |  |  |  |  |  |
| How many relatives do you feel close to such that you could call on them for help? |  |  |  |  |  |  |

End of Block: Lubben Social Network Scale 1

Start of Block: Lubben Social Network Scale 2

Q26
The following questions ask about friendships:

|  | Never | One | Two | Three or Four | Five Thru Eight | Nine or More |
| --- | --- | --- | --- | --- | --- | --- |
| How many of your friends do you see or hear from at least once a month? |  |  |  |  |  |  |
| How many friends do you feel at ease with that you can talk about private matters? |  |  |  |  |  |  |
| How many friends do you feel close to such that you could call on them for help? |  |  |  |  |  |  |

End of Block: Lubben Social Network Scale 2

Start of Block: Subjective Cognitive Function (SCF)

Q27 The following questions will ask about changes in your daily activities and memory.

|  | Yes | No |
| --- | --- | --- |
| Do you have more trouble than usual remembering recent events? |  |  |
| Do you have more trouble than usual remembering a short list of items, such as a shopping list? |  |  |
| Do you have trouble remembering things from one second to the next? |  |  |
| Do you have any difficulty in understanding things or following spoken instructions? |  |  |
| Do you have more trouble than usual following a group conversation or a plot in a TV program due to your memory? |  |  |
| Do you have trouble finding your way around familiar streets? |  |  |

End of Block: Subjective Cognitive Function (SCF)

Start of Block: Support

Display This Question:

If Do you have hearing loss? = Yes

Q28 In what ways do you feel you could be better supported as a person with hearing loss?
*Please select all that apply.*

- Greater Access to Hearing Technology
- Support Groups
- Educational Material on Hearing Loss
- Stress Reduction Information
- Listening Trainings
- Information on Meditation
- Information about Communication Strategies
- Other ________________________________________________

End of Block: Support
